# Maximizing theoretical and practical storage capacity in single-layer feedforward neural networks

**DOI:** 10.3389/fncom.2025.1646810

**Published:** 2025-08-25

**Authors:** Zane Z. Chou, Jean-Marie C. Bouteiller

**Affiliations:** ^1^Department of Biomedical Engineering, Viterbi School of Engineering, University of Southern California, Los Angeles, CA, United States; ^2^Institute for Technology and Medical Systems (ITEMS), Keck School of Medicine, University of Southern California, Los Angeles, CA, United States; ^3^Center for Artificial Intelligence and Quantum Computing in System Brain Research (CLARA), Prague, Czechia; ^4^International Neurodegenerative Disorders Research Center (INDRC), Prague, Czechia

**Keywords:** neural network, memory capacity, data-efficient AI, sustainable AI, constructive algorithms

## Abstract

Artificial neural networks are limited in the number of patterns that they can store and accurately recall, with capacity constraints arising from factors such as network size, architectural structure, pattern sparsity, and pattern dissimilarity. Exceeding these limits leads to recall errors, eventually leading to catastrophic forgetting, which is a major challenge in continual learning. In this study, we characterize the theoretical maximum memory capacity of single-layer feedforward networks as a function of these parameters. We derive analytical expressions for maximum theoretical memory capacity and introduce a grid-based construction and sub-sampling method for pattern generation that takes advantage of the full storage potential of the network. Our findings indicate that maximum capacity scales as (*N*/*S*)^*S*^, where N is the number of input/output units and S the pattern sparsity, under threshold constraints related to minimum pattern differentiability. Simulation results validate these theoretical predictions and show that the optimal pattern set can be constructed deterministically for any given network size and pattern sparsity, systematically outperforming random pattern generation in terms of storage capacity. This work offers a foundational framework for maximizing storage efficiency in neural network systems and supports the development of data-efficient, sustainable AI.

## 1 Introduction

**Figure 1:**
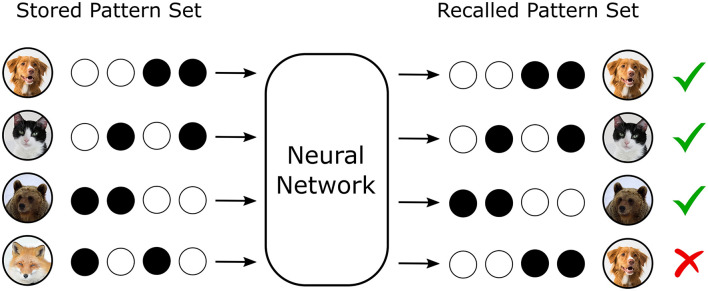
Neural networks can store and recall information. In a recall task where the desired output pattern is identical to the input pattern, several patterns can be stored and recalled without error. However, if the memory storage capacity of the network is exceeded, interference between the stored patterns results in failed recall.

Artificial neural networks (ANNs) are increasingly recognized for their capacity to serve as memory systems capable of storing, retrieving, and restructuring structured information (Hornik et al., 1989; Kohonen, 1972). In both biological and artificial systems, memory efficiency—the metric characterizing the quantity and fidelity with which information can be retained and recalled—has become a central concern due to constraints on computational cost, scalability, and energy consumption in AI applications (Horowitz, 2014; Strubell et al., 2019; Schwartz et al., 2020). These concerns are particularly acute in settings requiring continual learning, where new information must be assimilated without catastrophic interference or forgetting of previously acquired knowledge (Figure 1), a problem endemic to current machine learning paradigms (French, 1999; McCloskey and Cohen, 1989; Kirkpatrick et al., 2017).

The challenge of maximizing memory capacity within neural systems intersects with the core goals of the field of sustainable AI. Specifically, the need to optimize computational systems for recall accuracy and memory retention must be balanced against energy usage, data movement, and hardware limitations, especially in embedded or neuromorphic contexts (Zheng, 2025). While traditional ANN architectures typically require frequent retraining and substantial computational overhead, biological neural systems exhibit exceptional memory efficiency using sparse, distributed representations and local plasticity mechanisms. These characteristics have inspired several neuromorphic computing platforms that aim to replicate such properties in hardware, emphasizing energy efficiency, local processing, and event-driven computation (Fountain and Merkel, 2020; Pawlak and Howard, 2025).

Classical models of associative memory, such as the Hopfield network, define storage capacity as the maximum number of patterns that can be reliably retrieved above a set error rate (Hopfield, 1982). Early theoretical studies, particularly by (Gardner 1987); (Gardner and Derrida 1988), employed statistical physics to derive upper bounds on storage in perceptrons and recurrent architectures under idealized conditions. These seminal studies primarily focused on how storage capacity scaled with the size of the network and identified various closed-formed expressions for capacity bounds in different types of networks. More recent work (Fusi, 2024) has focused on overcoming catastrophic forgetting in continual learning scenarios (French, 1999; Parisi et al., 2019), leading to approaches such as synaptic regularization (Kirkpatrick et al., 2017), experience replay, and dynamic architectural expansion (Rusu et al., 2022). However, these strategies frequently introduce additional memory and energy costs, and few explicitly address the representational constraints that determine how many distinct patterns can be stored without degradation (Krauth and Mézard, 1989).

**Figure 2:**
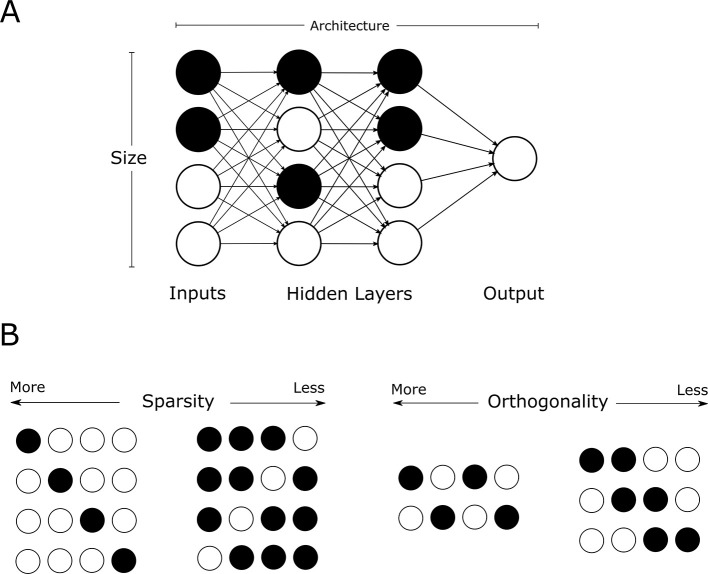
Variables that determine storage capacity of a network. **(A)** Network parameters. **(B)** Pattern representation.

In this context, sparsity and pattern orthogonality have emerged as critical determinants of storage capacity (Nadal and Toulouse, 1990) (Figure 2). Sparse representations, defined as a low fraction of active input/output units, are known to reduce interference between stored items and improve capacity in both artificial and biological systems (Kanerva, 1988; Olshausen and Field, 1996). Neuromorphic approaches extend this insight by implementing sparse, event-driven processing in hardware to reduce energy consumption and latency. Event-driven spiking neural networks (SNNs), for example, exploit sparse activity patterns to perform memory-related tasks at ultra-low power levels, a key advantage for implantable and edge-based AI systems (Schwartz et al., 2020; Pawlak and Howard, 2025). Pattern differentiability is known to provide a tradeoff in which more orthogonal patterns provide a lower risk of interference and recall error but result in lower capacities for the same network sizes as there are a fewer candidate patterns that maintain such high orthogonality. However, unlike with network size, the explicit dependence of capacity on sparsity and orthogonality is not well characterized (Krauth and Mézard, 1989).

This study proposes a novel constructive framework for quantifying and attaining the maximum pattern storage capacity of single-layer binary feedforward neural networks. This framework explicitly links representational parameters such as network size, pattern sparsity, and minimum pattern differentiability to closed-form capacity expressions derived via combinatorial analysis. In addition, we introduce an algorithm to construct the full set of storable patterns through matrix saturation and structured sub-sampling. This approach is not only analytically grounded, but also energy- and memory-efficient, as it avoids network retraining or modification of previously stored weights. The method consistently reaches the derived theoretical maximum, surpassing random generation strategies that suffer from pattern overlap and redundancy. It also provides a heuristic for optimizing pattern reorganization within a sub-optimally initialized network that cannot reach the theoretical maximum capacity.

## 2 Methods

**Figure 3:**
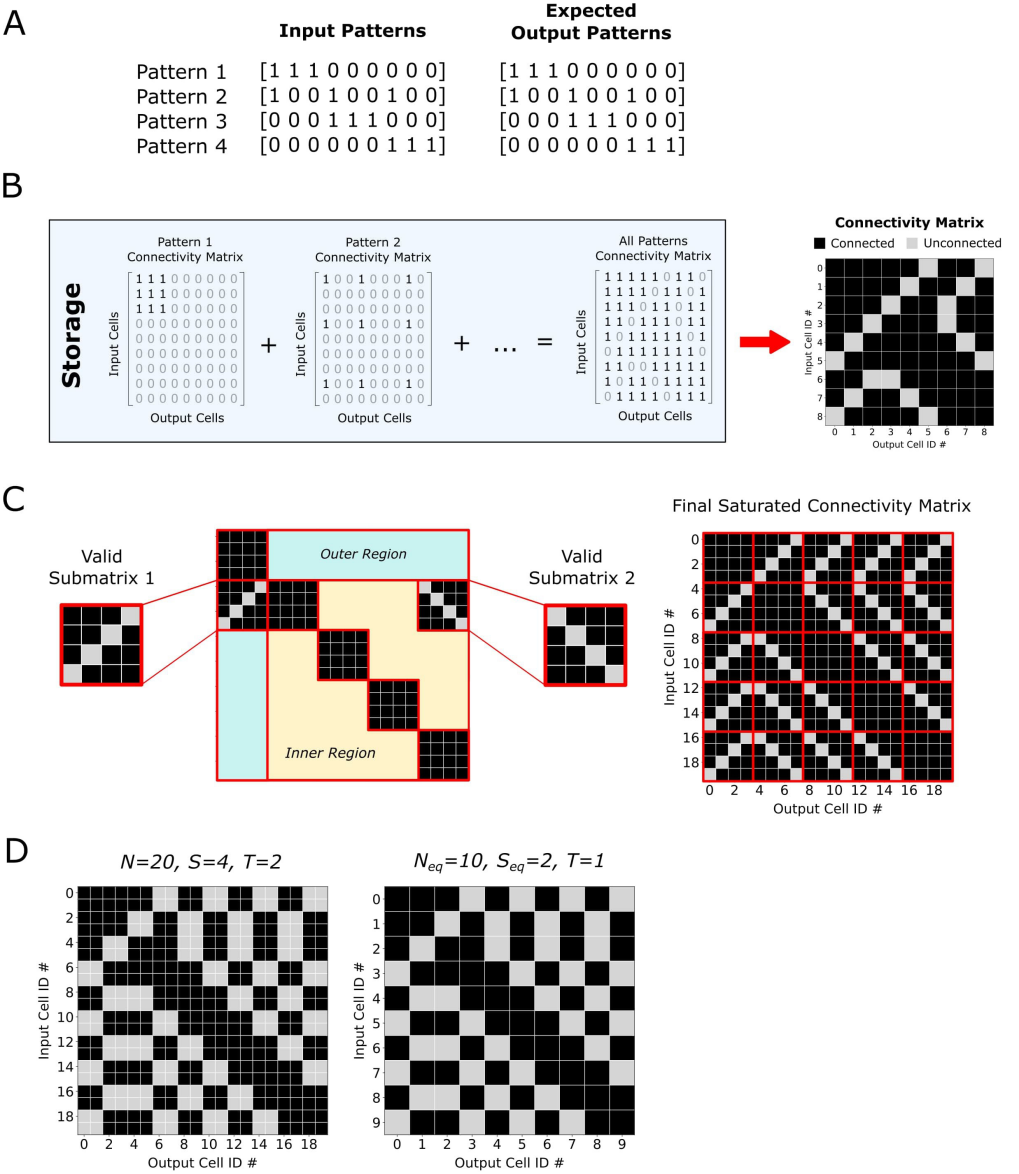
**(A)** For a given pattern set, the input and desired output patterns are defined as identical. **(B)** The connectivity matrix is constructed by superimposing the individual connectivity matrices defined by every stored pattern. **(C)** A fully saturated connectivity matrix can be modularly constructed from valid sub-matrices. For maximum threshold cases, valid sub-matrices contain one empty cell per row and column, and are appropriately repeated throughout the outer and inner regions of the connectivity matrix. Patterns that can be recalled without error are the patterns that would not cause further connections to be made. Thus all valid patterns that can be recalled without error can be obtained by sub-sampling this saturated matrix. **(D)** The saturation matrix for different threshold levels can be determined by using a smaller equivalent matrix at the maximum threshold level.

Our preliminary analysis was conducted with a simplified model of a neural network consisting of a single-layer, fully connected, feedforward architecture with binary weights. The network contains *N* input and *N* output neurons, where the cued input patterns are assumed to be identical to the corresponding target output patterns, both represented by binary vectors (Figure 3A). Let *S* denote the number of active elements in each input pattern (i.e., pattern sparsity), and *D* represent the minimum required Hamming distance between any two stored patterns, which ensures distinguishability during recall. An activation threshold *T* is used to determine the firing of output neurons based on the weighted sum of incoming signals.

To evaluate capacity, a set of *P* input patterns is incrementally stored in the network. For each individual pattern, a connectivity matrix containing ones for all connections between the active input cells and their identical output cells can be constructed. The total connectivity matrix is created by superimposing all individual matrices and clipping the result to remain binary (Figure 3B). During recall, the input pattern is multiplied by the connectivity matrix, and output activation is determined by comparing the resulting sums against the threshold *T*. Patterns are deemed successfully recalled if the reconstructed output exactly matches the original pattern.

Patterns are initially generated randomly, and the storage process continues until no additional pattern can be added without inducing recall errors. This exhaustive approach frequently fails to reach theoretical capacity due to the stochastic nature of random generation and its susceptibility to early pattern correlations, which lead to premature saturation of the connectivity matrix.

To mitigate this limitation, a heuristic metric termed cross-pattern activation (CPA) was introduced. CPA measures the summed pre-threshold activations of output neurons across all stored patterns and is used to guide the selection of new candidate patterns. While CPA provided some improvement by minimizing interference during pattern addition, it ultimately proved ineffective in scenarios where the initial pattern distribution was suboptimal or where all candidate patterns yielded equivalent CPA values. Notably, the ideal capacity-achieving state is characterized by a fully saturated connectivity matrix in which the CPA values for all non-pattern output cells lie just below the activation threshold (i.e., equal to *(T - 1)*).

An alternative strategy was devised to instead start from a predefined connectivity matrix that would be deterministically initialized in this saturated state. A grid-based decomposition approach is employed that satisfies the constraints at maximum threshold, where *D* = 1, and *T* = *S*−1. First, we define a set of *B* = *N*/*S* orthogonal basis patterns that each activate a unique subset of S inputs. These basis patterns naturally partition the connectivity matrix into a *B*×*B* grid of *S*×*S* sub-matrices, where the basis patterns fully saturate the diagonal sub-matrices. Future patterns added to the pattern set must differ from these basis patterns by D active cells and thus each sub-matrix not on the diagonal must have at least one unconnected cell per row and column. Furthermore, to provide the structural regularity that preserves the maximum number of storable patterns derivable from the saturated connectivity matrix, all sub-matrices along the outer boundary should maintain a consistent internal orientation. Similarly, sub-matrices in the interior region of the matrix should share a uniform orientation, which may, but need not, match that of the boundary sub-matrices (Figure 3C). Other optimal connectivity matrices can be obtained by permuting the order of the cells within a sub-matrix and then performing the appropriate permutation across all sub-matrices that share its orientation.

**Figure 4:**
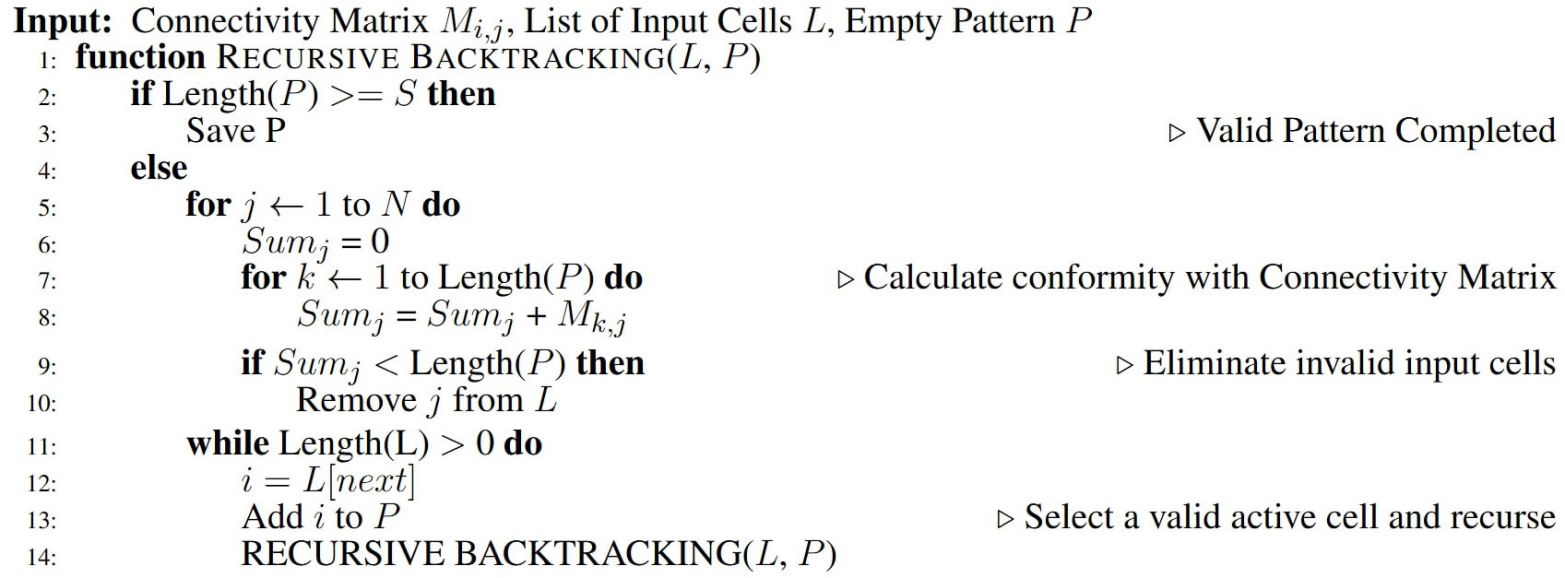
Patterns are deterministically generated from the saturated connectivity matrix using a recursive backtracking algorithm.

Valid patterns are then generated by recursively sampling combinations of S active cells that conform to the fixed matrix. The sub-sampling algorithm, presented in pseudocode form in Figure 4, involves a backtracking approach that eliminates combinations of active cells that would violate the requirements that some elements in the connectivity matrix must remain unconnected, and thus ensures that each new pattern does not alter the matrix and avoids conflicts with previously stored patterns.

The total number of such patterns, corresponding to the network's theoretical maximum capacity, is derived from combinatorial analysis and shown to be:


(1) $$C=\sum_{i=0}^{\frac{N}{S}-1}\sum_{j=0}^{S}\left(\begin{array}{c} S\\ j \end{array}\right)i^{j}.$$

Each iteration of the summation enumerates the unique combinations in which the *i*-th block can contain *j* active elements, with the rest of the *S*−*j* active elements being distributed among the other blocks. Simplifying using the binomial theorem yields an expression for the storage capacity of the network


(2) $$\begin{array}{l} C={\left(N/S\right)}^{S}, \end{array}$$

under the assumption that threshold is set at the maximum *T*_max_ = *S*−1. For thresholds where *T* = *S*−*D* and *D*>1, an equivalent network transformation is performed by redefining the network size as *N*_eq_ = *N*/*D* and sparsity as *S*_eq_ = *S*/*D*, thus preserving the saturation condition (Figure 3D).

## 3 Results

To evaluate the theoretical framework and validate the constructive approach to capacity maximization, a series of simulations were conducted across a range of network sizes and pattern representation parameters. The goal was to determine how storage capacity scales with network dimensions, sparsity levels, and pattern differentiability constraints, and to assess the effectiveness of different pattern generation strategies under these conditions.

**Figure 5:**
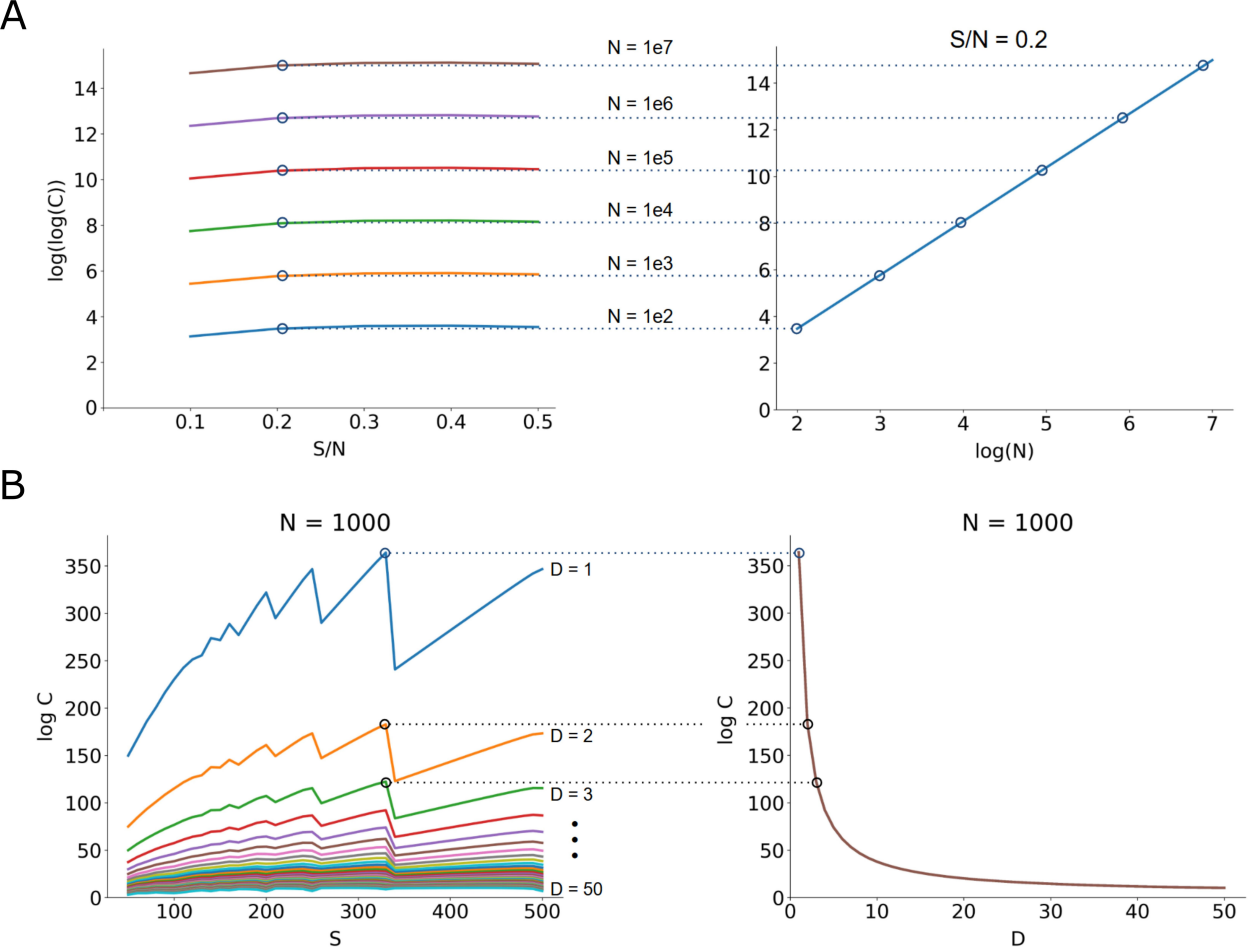
Maximum storage capacity of a network increases with network size. **(A)** For increasing network size, while keeping the relative pattern sparsity constant, capacity increases logarithmically. **(B)** When keeping network size constant, for varying pattern differentiability, the ideal pattern sparsity occurs around N/3. The maximum capacity achievable for the network is inversely related to differentiability.

First, the relationship between network size *N* and maximum memory capacity was investigated. Networks with sizes ranging from 10^2^ to 10^7^ neurons were analyzed, and the number of storable, non-overlapping patterns was computed as a function of pattern sparsity *S*, expressed as a fraction of *N*. When *(S/N)* was held constant, the observed memory capacity exhibited a logarithmic increase with respect to *N*, consistent with the theoretical prediction derived from combinatorial analysis (Figure 5A). This trend reinforces the principle that, under fixed sparsity, capacity grows superlinearly with network size, in agreement with findings from Gardner.

To explore the trade-offs between sparsity and pattern differentiability, further simulations were performed with a fixed network size while systematically varying the minimum Hamming distance *D* between stored patterns and the sparsity *S*. The results revealed a consistent optimal storage configuration near *S*≈*N*/3 with deviations above or below this threshold leading to diminished capacity due to either excessive overlap (high *S*) or insufficient representational density (low *S*) (Figure 5B). As expected, storage capacity was found to be inversely proportional to *D*, indicating that stricter pattern separation constraints reduce the number of storable patterns. Since higher *D* effectively raises the minimum activation threshold *T*, this result aligns with theoretical expectations. Importantly, although higher thresholds consistently improve storage capacity across varying sparsity levels in our proposed framework, this approach prioritizes one specific neural function, i.e., pattern separation. Future studies exploring pattern completion mechanisms would likely reveal a different relationship—where optimal performance requires balancing threshold levels against the system's capacity to accurately reconstruct degraded input patterns.

**Figure 6:**
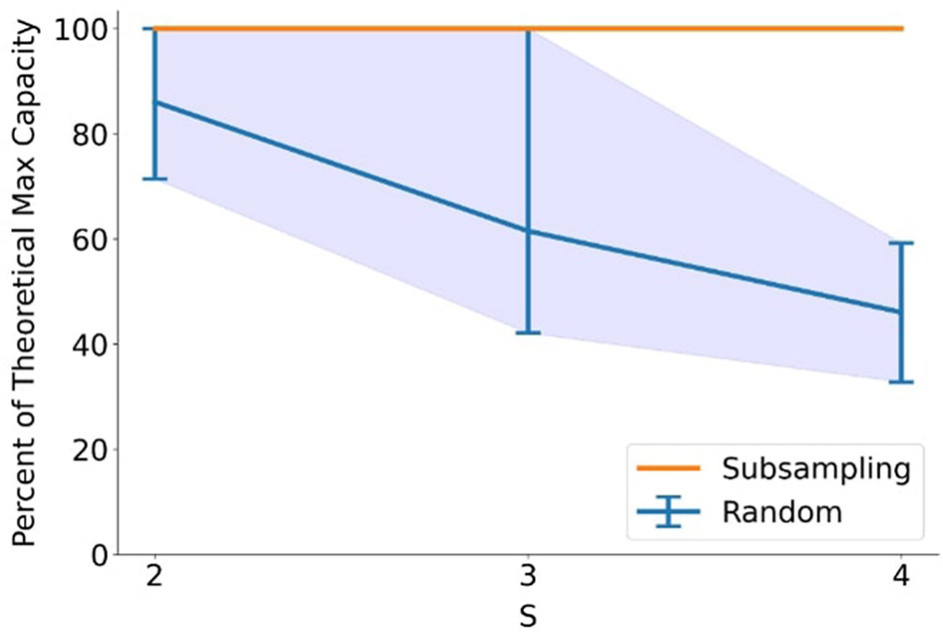
Pattern generation using sub-sampling from the connectivity matrix is able to consistently achieve the maximum theoretical capacity for the network at various different network sizes and pattern sparsities, thus outperforming purely random pattern generation. Results were averaged for values of *N* equal to small integer multiples of *S* between 12 and 24. Maximum and minimum variability of random trials are indicated by the envelope.

The practical implications of the constructive algorithm were evaluated by comparing two pattern generation strategies: (1) stochastic random generation with sequential pattern insertion, and (2) deterministic sub-sampling from a saturated connectivity matrix configured to preserve threshold conditions. The latter approach consistently achieved 100% of the theoretical maximum capacity across all tested values of *N, S*, and *D*, where *N* was set to small integer multiples of S, whereas random generation exhibited high variance and frequent early saturation due to unstructured pattern correlations (Figure 6). These results underscore the inefficiency of trial-and-error memory formation and demonstrate the clear advantage of structure-aware pattern synthesis in maximizing information density, an observation that aligns with proposed benefits of memory consolidation during sleep (Diekelmann and Born, 2010).

To assess the feasibility of memory reorganization in suboptimal networks, additional experiments were conducted wherein a network was first initialized with a random pattern set whose capacity fell below the theoretical maximum. An optimal pattern set was then generated using the proposed algorithm, and the distance between the two sets was quantified by computing the total Hamming distance between best-matched pattern pairs. Using this metric, the minimum computational effort required to transform the suboptimal set into the optimal one was estimated via two components: (1) bit-flipping operations required to convert individual patterns, and (2) pattern deletions necessary to eliminate irreconcilable overlap. The cost of reorganization was computed as the sum of flipped bits and deleted patterns required to reach the maximum storable configuration. The primary goal of this investigation was to determine the optimal balance between the two reorganization strategies, and as such the constant computational cost to evaluate the viability of each pattern within the randomly generated pattern set was excluded from this analysis.

**Figure 7:**
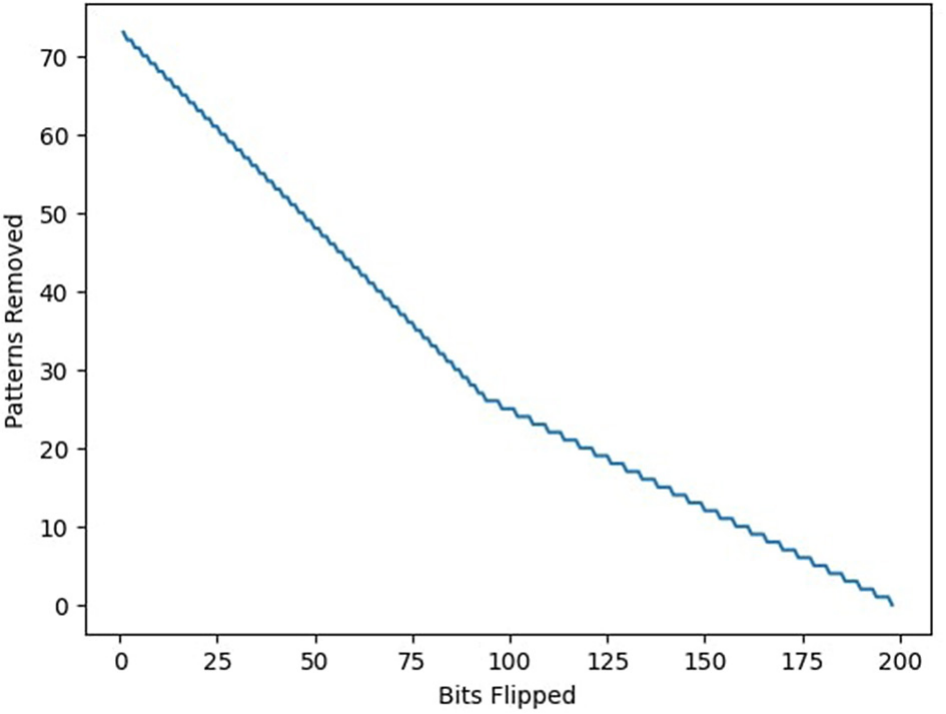
Starting from a suboptimal state, the amount of work needed to achieve the maximum theoretical capacity is compared. Work can be performed by either flipping a bit or removing an erroneous pattern entirely, which takes *S* bits of work for pattern size *S*. The minimum amount of work to reach maximum capacity can be determined by minimizing the sum of bits flipped and bits removed.

The transformation trajectory, plotted as a function of incremental bit-flipping interventions, revealed a nonlinear trade-off between bit-level edits and pattern removals (Figure 7). This analysis provides a quantitative measure of memory plasticity in fixed-architecture systems and suggests an efficient route to capacity recovery without full retraining. From a sustainability perspective, this capacity restoration strategy is significant: it reduces the computational overhead associated with complete reinitialization or expansion, and thus aligns with the principles of energy-efficient memory maintenance in embedded and neuromorphic systems (Pawlak and Howard, 2025).

Collectively, these results validate the theoretical predictions and demonstrate that the proposed constructive method offers both optimal storage performance and operational efficiency. In contrast to stochastic methods that rely on probabilistic insertion and fail to guarantee convergence to maximal configurations, the sub-sampling algorithm provides a deterministic and scalable route to capacity maximization, making it particularly suitable for applications in real-time, low-power environments.

## 4 Discussion

The findings of this study represent both a theoretical and practical contribution to the understanding of memory capacity in constrained neural systems. By deriving a closed-form expression for maximum pattern storage in binary feedforward networks, we provide a formal characterization of capacity that integrates both structural and representational constraints into a predictive framework. Unlike prior probabilistic or heuristic approaches, the constructive method introduced here achieves theoretical capacity deterministically, without retraining or modification of existing weights. This makes the approach particularly suitable for deployment in settings where computational efficiency and energy conservation are paramount.

Importantly, the principles developed herein have broader implications for the design of neuromorphic systems. Constructive memory optimization aligns with the goals of sustainable AI by enabling real-time, low-power storage and recall in constrained environments, such as implantable neurotechnologies where memory and energy budgets are tightly limited. Recent advances in neuromorphic platforms such as Loihi and SpiNNaker have demonstrated the feasibility of deploying SNN-based algorithms for continual learning and efficient memory handling (Davies et al., 2018; Furber et al., 2014). By contributing both theoretical and algorithmic tools to this effort, this work addresses an unmet need at the intersection of memory capacity, representation theory, and sustainable AI.

The capacity-achieving constructive algorithm developed in this study complements existing neuromorphic paradigms by enabling dense and non-overlapping memory storage without resorting to retraining or architectural expansion. This is particularly relevant in systems that must support continual learning across a long time horizon, such as adaptive brain-computer interfaces or closed-loop implants. In such settings, the capacity to reorganize stored patterns retroactively and achieve near-optimal usage of the network's representational space offers a compelling alternative to traditional retraining, which is both computationally and energetically expensive. As shown in this study, even suboptimal memory configurations can be transformed into near-optimal ones through selective bit-flipping and pattern pruning, minimizing both energy expenditure and memory reinitialization.

This also aligns with recent calls for more sustainable AI, where algorithmic advances must account not only for accuracy or generalization but also for operational efficiency, particularly under constraints of size, latency, and power (Horowitz, 2014; Strubell et al., 2019; Schwartz et al., 2020). In the context of neuromorphic implants, where thermal dissipation, battery life, and biocompatibility impose hard limits on computation, energy-efficient memory management becomes essential. Notably, the principles of sparse, structured encoding and interference minimization described here are also mirrored in biological systems, where memory traces are believed to be distributed across sparse assemblies and differentiated by temporal and spatial coding strategies (Olshausen and Field, 1996; Pawlak and Howard, 2025).

Furthermore, this study contributes to ongoing discussions around adaptive learning in dynamic environments. Biological systems continually modify their internal representations in response to changing stimuli, while maintaining robust memory of prior experiences, which is a capability still elusive in artificial systems. Although spiking neural networks (SNNs) have shown promise in this direction, many current learning algorithms for SNNs rely on stochastic gradient descent or plasticity rules such as spike-timing-dependent plasticity (STDP), which, while biologically inspired, often fall short of providing memory guarantees under structural constraints (Indiveri and Liu, 2015; Subramoney et al., 2024; Zenke and Laborieux, 2025). By contrast, the approach described here establishes a constructive route to capacity optimization that can be implemented independently of learning rules, offering a complementary strategy for memory consolidation in adaptive systems.

Future work will seek to generalize this framework to multi-layer and recurrent architectures, which are common in both cortical models and neuromorphic implementations, as well as to implement non-binary weights and investigate how synaptic updates during learning change the patterns that can be derived from a non-binary weight matrix. Possible approaches could involve representing patterns generated from non-binary weights as probabilistic rather than deterministic or implementing a dynamic threshold on the weight matrix that varies for different input cells. Additionally, extending the model to incorporate noise tolerance and strike a balance between pattern completion and separation would further bridge the gap between idealized theoretical capacity and real-world neuromorphic deployment. Such extensions are necessary to align the theoretical insights of this study with practical challenges encountered in biological environments, including variability of neural responses, non-stationary inputs, and the need for real-time learning and recall.

One promising direction to look to is Hyperdimensional Computing (HDC), which uses randomized, distributed hypervectors and compositional operations (e.g., bundling and binding) to store and process information in a robust and scalable manner (Kanerva, 2009). HDC has been shown to be a powerful method of pattern representation that increases memory capacity of networks (Frady et al., 2018). However, in contrast to our constructive approach that deterministically generates the maximum number of unique, separable patterns, HDC tends to prioritize pattern completion and the ability to recall stored patterns even with noisy or degraded inputs. HDC could provide a natural direction expand our work by balancing the tradeoff between pattern separation and pattern completion, while also potentially providing a solution for generalizing the connectivity matrix with non-binary weights or by representing the connectivity matrix as an n-dimensional cube, with the number of dimensions corresponding to the number of blocks, instead of a 2D matrix.

In summary, by offering a scalable, energy-efficient, and constructive method for maximizing memory capacity, this work advances the theoretical foundation and practical viability of sustainable neural architectures. The proposed approach is especially suited to neuromorphic applications in which future intelligent systems must not only learn effectively but also remember efficiently within the constraints set by their limited resources.

## Data Availability

The datasets presented in this study can be found in online repositories. The names of the repository/repositories and accession number(s) can be found here: https://github.com/zzhchou/memory_capacity.
